# Nanoparticle-based approaches for treating restenosis after vascular injury

**DOI:** 10.3389/fphar.2024.1427651

**Published:** 2024-10-24

**Authors:** Liangfeng Zhao, Liuliu Feng, Rong Shan, Yue Huang, Li Shen, Mingliang Fan, Yu Wang

**Affiliations:** ^1^ School of Health Science and Engineering, University of Shanghai for Science and Technology, Shanghai, China; ^2^ Department of Cardiology, Shidong Hospital Affiliated to University of Shanghai for Science and Technology, Shanghai, China; ^3^ Department of Cardiology, Zhongshan Hospital, Shanghai Institute of Cardiovascular Diseases, Fudan University, Shanghai, China

**Keywords:** nanoparticles, vascular restenosis, controlled release, targeted drug delivery, water solubility

## Abstract

Percutaneous coronary intervention (PCI) is currently the main method for treating coronary artery stenosis, but the incidence of restenosis after PCI is relatively high. Restenosis, the narrowing of blood vessels by more than 50% of the normal diameter after PCI, severely compromises the therapeutic efficacy. Therefore, preventing postinterventional restenosis is important. Vascular restenosis is mainly associated with endothelial injury, the inflammatory response, the proliferation and migration of vascular smooth muscle cells (VSMCs), excessive deposition of extracellular matrix (ECM) and intimal hyperplasia (IH) and is usually prevented by administering antiproliferative or anti-inflammatory drugs through drug-eluting stents (DESs); however, DESs can lead to uncontrolled drug release. In addition, as extracorporeal implants, they can cause inflammation and thrombosis, resulting in suboptimal treatment. Therefore, there is an urgent need for a drug carrier with controlled drug release and high biocompatibility for *in vivo* drug delivery to prevent restenosis. The development of nanotechnology has enabled the preparation of nanoparticle drug carriers with low toxicity, high drug loading, high biocompatibility, precise targeting, controlled drug release and excellent intracellular delivery ability. This review summarizes the advantages of nanoparticle drug carriers for treating vascular restenosis, as well as how nanoparticles have improved targeting, slowed the release of therapeutic agents, and prolonged circulation *in vivo* to prevent vascular restenosis more effectively. The overall purpose of this review is to present an overview of nanoparticle therapy for vascular restenosis. We expect these findings to provide insight into nanoparticle-based therapeutic approaches for vascular restenosis.

## 1 Introduction

Cardiovascular diseases (CVDs) and their complications are among the leading causes of human mortality (Soumya and Raghu, 2023). About 45%–75% of the causes of CVD are related to atherosclerosis (Francula-Zaninovic and Nola, 2018). Atherosclerosis is the primary contributor to coronary artery atherosclerotic disease, cerebral infarction, and peripheral vascular disease. Fatty streaks in the arterial wall gradually develop into the characteristic plaques of atherosclerosis. The clinical consequences of these plaques depend on their location and on the degree and velocity of vessel occlusion (Libby, 2021). The disease has a latent period of many years and often coexists in multiple vascular beds. The acute rupture of atherosclerotic plaques can lead to local thrombosis, resulting in partial or complete occlusion of the affected artery, which can in turn cause acute myocardial infarction and other pathologies. When atherosclerotic plaque severely obstructs a vessel (stenosis greater than 75%), angioplasty is usually necessary, as the classic clinical approach for eliminating plaque and restoring blood supply (Wang et al., 2022). However, the problem of vascular restenosis after interventional procedures has seriously hampered the development of vascular interventions.

Vascular restenosis usually occurs within 3–8 months of revascularization. When the lumen of a blood vessel narrows again to less than 50% of its normal diameter after interventional treatment, this is called vascular restenosis (Kibos et al., 2007). Restenosis is not a random phenomenon, and patients with certain diseases, such as diabetes and hypertension, have an increased probability of developing vascular restenosis (Nakamura et al., 2024; Takano et al., 2024). Although we do not have a clear understanding of the mechanisms, vascular restenosis is generally accepted to be associated with excessive proliferation and migration of vascular smooth muscle cells (VSMCs), excessive deposition of extracellular matrix (ECM), and intimal hyperplasia (IH) (Trepanier et al., 2023).

The prevention of vascular restenosis can be approached by addressing its causes. The main focus is to promote the re-endothelialization of blood vessels, inhibit vascular smooth muscle cell proliferation and migration, and ameliorate inflammation, platelet aggregation and collagen degradation. Recent advances in nanomedicine and targeted drug therapies have driven the development of treatments for vascular restenosis. Moreover, this study introduces new avenues for the treatment of vascular restenosis. Nanoparticles, which are particles with size less than 1,000 nm, are a widely used type of delivery platform (Medina et al., 2007; Strambeanu et al., 2015). Currently, drugs used for the treatment of vascular restenosis mainly include nucleic acids, proteins, small-molecule drugs and gas signaling molecules (Melnik et al., 2022). However, the safety and targeting potential of these therapeutic agents are poor. To overcome some of the shortcomings of these therapeutic agents, such as poor solubility, low stability, weak targeting, and potential biotoxicity, we can encapsulate therapeutic agents into nanoparticles and then modify the surface of the nanoparticles (Mitchell et al., 2021). Thus, nanoparticles can be used as an efficient carrier platform for the *in vivo* targeted delivery of therapeutic drugs for vascular restenosis.

This review first describes the pathophysiology of vascular restenosis and then briefly describes the main strategies to combat restenosis after vascular injury in relation to the pathophysiological process. Commonly used drug-carrying nanoparticles and modalities are introduced, and the applications and advantages of nanoparticles in vascular restenosis are summarized. In addition, we discuss how nanodelivery strategies can be applied to treat vascular restenosis. In the main advantages of the increased blood circulation time and vascular restenosis targeting ability of the nanoparticles after drug loading, as well as the effective binding and release of therapeutic agents. Finally, future prospects for preventing and treating vascular restenosis are discussed.

## 2 Pathophysiology of vascular restenosis after vascular injury

The walls of arterial and venous vessels are divided, from the inside out, into the tunica intima (or intima), tunica media (or media), and tunica adventitia (or extima). Among these layers, the tunica intima is the thinnest, consists mainly of endothelial cells, and is subdivided from the inside out into three layers—the endothelium, subendothelium, and internal elastic membrane. The tunica media consists of an elastic membrane, smooth muscle fibers, and connective tissue, the thickness and composition of which vary considerably from vessel to vessel. The elastic membrane and elastic fibers of the tunica media retract dilated vessels, and collagen fibers maintain their tone. Vascular smooth muscle fibers consist mainly of VSMCs and exhibit two functional states or phenotypes. Those with predominantly synthetic and secretory functions are called synthetic phenotypes, and those with predominantly contractile functions are called contractile phenotypes. The tunica adventitia consists of loose connective tissue and contains fibroblasts that can repair it.

The surgically induced inflammatory response may be the primary cause of restenosis. We currently believe that endovascular interventional treatment inevitably causes vascular endothelial injury, which is the starting point for a cascade of activity involving cells and tissues at the injury site. When the area of intimal damage is small, there is increased secretion of proliferative growth factors from endothelial cells (ECs), which promotes repair of the intima. In this case, the tunica intima will not become inflamed. When the damage to the tunica intima is severe, the ECs are no longer able to fully restore the integrity of the tunica intima, which can lead to an inflammatory response. The inflammatory response in turn leads to the proliferation and migration of VSMCs, the formation of ECM, and IH, ultimately leading to vascular restenosis (Inoue et al., 2011; Lyle and Taylor, 2019).

**FIGURE 1:**
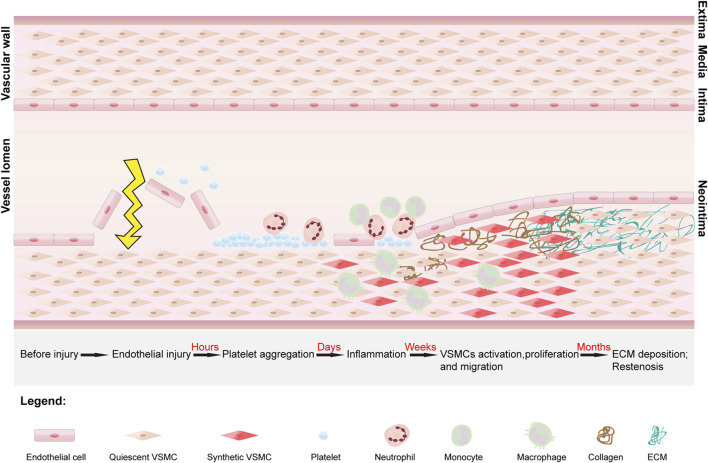
The stages of development of vascular restenosis and the structure of the vessel. Vascular restenosis begins with intimal injury, after which platelets begin to aggregate and adhere at the site of injury. The platelets release mitogenic growth factors, which recruit leukocytes, neutrophils, and macrophages to the site, ultimately converting vascular smooth muscle cells (VSMCs) from a quiescent phenotype to a synthetic phenotype. Under the influence of growth factors and inflammatory mediators, synthetic VSMCs proliferate and migrate into the subendothelial space. In addition, synthetic VSMCs secrete collagen. Under continuous stimulation by growth factors, there is an imbalance between collagen synthesis and degradation, leading to excessive deposition of the extracellular matrix (ECM). Ultimately, the proliferation and migration of VSMCs and the excessive deposition of ECM lead to the development of vascular restenosis.

The main cause of vascular restenosis is IH, which is a complex biological cellular response process controlled by a range of cells, including platelets, macrophages, neutrophils, leukocytes, VSMCs and endothelial cells. Beginning with intimal injury, this process develops over time and can be divided into three main phases: the acute, neutral and chronic phases (Melnik et al., 2022) (Figure 1).

In the acute phase, immediately after intimal injury, platelets aggregate at the site of injury and adhere primarily to the subendothelial matrix. In the damaged endothelium, large amounts of von Willebrand factor (vWF) are deposited on the subendothelial surface at the site of vascular damage (Su et al., 2014). Platelet surface receptors bind to vWF, leading to platelet deposition at the site of vascular injury. In addition, the secretion of VSMC proliferation inhibitors, such as nitric oxide (NO), decreases, whereas the secretion of prothrombotic factors increases (Landzberg et al., 1997). In the IH environment, NO not only stimulates the proliferation of ECs and accelerates the restoration of the endothelial layer but also reduces inflammatory chemotaxis. A decrease in the mount of NO leads to the proliferation and migration of VSMCs (Ahanchi et al., 2007). Vascular endothelial growth factor (VEGF) plays an important role in endothelial healing and growth and can promote NO synthesis to inhibit vascular restenosis. The secretion of VEGF also decreases in the acute phase, which can delay endothelial healing and ultimately cause vascular restenosis (Hood et al., 1998; Luo et al., 1998). In addition, the secretion of platelet-derived growth factor (PDGF), mainly by platelets, is increased, and promotes the migration and proliferation of VSMCs, which in turn promotes vascular restenosis (Andrae et al., 2008; Xue et al., 2023).

During the neutral phase, a platelet-dominated coagulation cascade reaction begins, and this process lasts for several days after endothelial injury. The coagulation cascade leads to platelet activation and fibrin deposition, followed by the production of thrombin (a mitogen that promotes mitosis in VSMCs) (Chandrasekar and Tanguay, 2000). Platelets continue to release growth factors (GFs) during this process and recruit immune cells (leukocytes, macrophages, neutrophils, etc.) to aggregate into the injury area. In the presence of GFs, the VSMC phenotype changes from a quiescent contractile state to a synthetic state, resulting in proliferation (Jukema et al., 2011). The ECM is decomposed by PDGF, basic fibroblast growth factor (BFGF), and matrix metalloproteinases (MMPs), thereby promoting the migration of VSMCs into the subendothelium. At this time, the subendothelium consists mainly of dedifferentiated VSMCs (Melnik et al., 2022). In particular, transforming growth factor-β (TGF-β) secreted by activated platelets can activate multiple intracellular signaling pathways, accelerating the repair of VSMCs in the tunica intima. Moreover, TGF-β can promote the proliferation and dedifferentiation of VSMCs by binding to cell surface ligands, thereby phosphorylating intracellular smad 2/3 proteins. In addition, smad 2/3 can promote the proliferation of VSMCs by activating the extracellular signal-regulated kinase (ERK 1/2) pathway of the phosphorylated mitogen-activated protein kinase (MAPK) family (Suwanabol et al., 2011).

In the chronic phase, a period that extends primarily from the first week to several months after injury, VSMCs continue to proliferate in response to GFs, while the ECM is resynthesized (Osherov et al., 2011). Collagen is a major component of the ECM and is secreted by synthetic VSMCs. Overproduction of collagen leads to an imbalance between synthesis and degradation of the ECM. Eventually, excessive proliferation and migration of VSMCs and excessive deposition of ECM lead to vascular restenosis.

**FIGURE 2:**
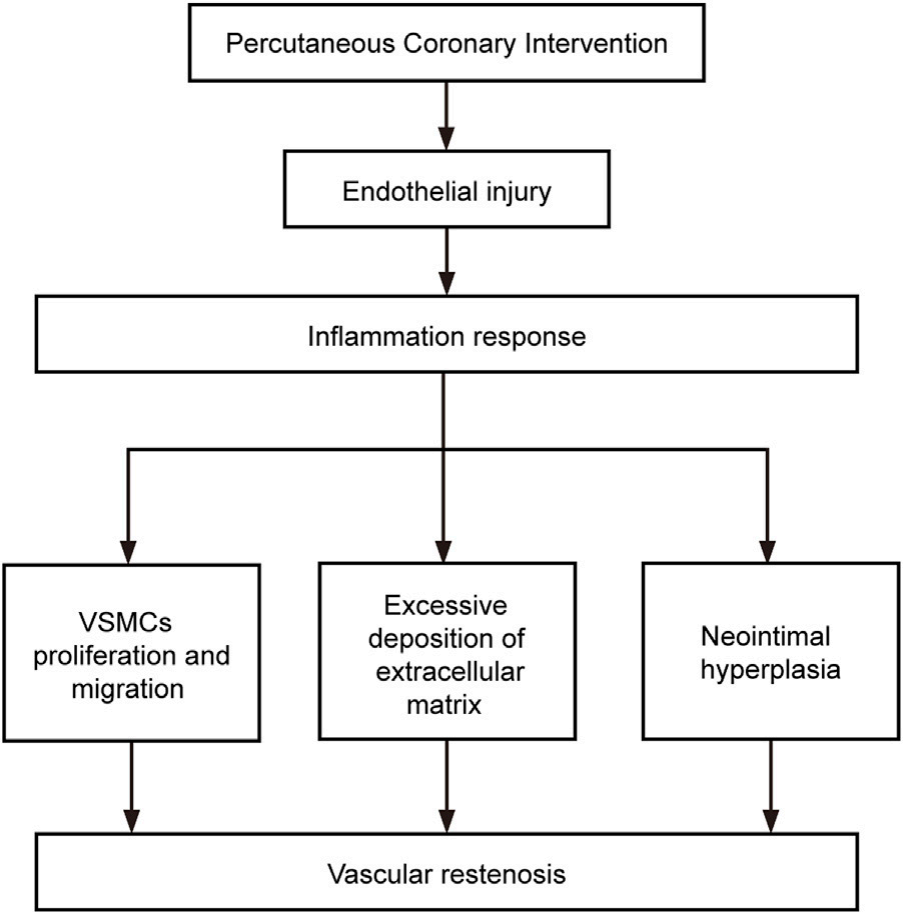
Factors involved in the development of restenosis. The flow chart shows the relationship between the components that influence the development of restenosis.

In the pathological process of vascular restenosis described above, intimal damage caused by surgery is the initiating factor leading to restenosis, the inflammatory response is the key step, and IH is the crucial factor causing vascular restenosis (Figure 2).

## 3 Primary strategies for preventing vascular restenosis after vascular injury

### 3.1 Promotion of vascular reendothelialization

The tunica intima of blood vessels consists mainly of tightly connected monolayer endothelial cells. The intact endothelium not only insulates the blood but also regulates vascular function. ECs secrete a variety of GFs, which regulate the inflammatory response, platelet adhesion, and proliferative behavior of VSMCs. Vascular endothelial injury is the initiating factor for vascular restenosis. Promoting vascular reendothelialization helps to block the occurrence of vascular restenosis. Currently, reendothelialization of blood vessels can be promoted by the local delivery of VEGF, the local delivery of drugs that promote reendothelialization, and the implantation of stents that attract endothelial cell adhesion and endothelial progenitor cell (EPC) adhesion (Kipshidze et al., 2004; Liu et al., 2017a; Xia et al., 2021; Kang et al., 2022; Hua et al., 2023).

### 3.2 Inhibition of vascular smooth muscle cell proliferation and migration

An important factor contributing to vascular restenosis is the proliferation and migration of VSMCs. Under normal physiological conditions, VSMCs in the vascular tunica media are highly differentiated and quiescent. They mainly maintain vascular tone, regulate vasoconstriction, and do not have the ability to proliferate or migrate. However, extensive endothelial damage can cause platelet aggregation and an inflammatory response. In addition, GFs, PDGF, BFGF, and MMPs are recruited to the site of vascular injury (Melnik et al., 2022). The combined action of these cytokines promotes the conversion of VSMCs from a quiescent contractile phenotype to an active synthetic phenotype and promotes the proliferation and migration of VSMCs, ultimately leading to vascular restenosis. Therefore, vascular restenosis can be prevented by inhibiting the proliferation and migration of VSMCs. Currently, vascular restenosis can be prevented by the local delivery of antiproliferative drugs, local delivery of genes, or coating stents with antiproliferative drugs in stents (Banai et al., 2005; Yang et al., 2008; Yang et al., 2011).

### 3.3 Inhibition of inflammatory response

Inflammatory responses are induced after extensive intimal injury and play an important role in the proliferation and migration of VSMCs. Targeting the inflammatory response to prevent restenosis is also an important strategy. Currently, the prevention of inflammation is carried out mainly by the local delivery of anti-inflammatory drugs and coating stents with anti-inflammatory drugs (Park and Yoo, 2006; Inoue et al., 2011).

### 3.4 Inhibition of platelet aggregation

Following vascular injury, platelets rapidly aggregate at the site of vascular injury and adhere to the subendothelial matrix. The platelets, in turn, recruit leukocytes, monocytes, macrophages, and neutrophils, among others, to aggregate at the site of vascular injury (Inoue et al., 2011). A complex series of biological cellular reactions then occur, ultimately leading to vascular restenosis. Therefore, inhibiting platelet aggregation can also prevent vascular restenosis. Currently, mainly oral antiplatelet drugs, including aspirin and clopidogrel, can be used to prevent vascular restenosis through local delivery to the location of vascular injury (Li et al., 2017).

### 3.5 Regulation of collagen synthesis and degradation

Synthetic VSMCs also overproduce collagen, which is a major component of the ECM (Batchelor et al., 1998). An imbalance in ECM synthesis and degradation leads to vascular restenosis, and in the chronic phase of vascular restenosis, the subendothelial layer consists predominantly of ECM (Chung, 2004). Therefore, the regulation of collagen synthesis and degradation is also an important direction for preventing vascular restenosis.

## 4 Nanotechnology and nanoparticle carriers

Nanoparticles (NPs) are substances with a particle size between 1 and 1,000 nm (Medina et al., 2007). Sometimes their size can reach several hundred nanometers (Pillai, 2019). In recent years, nanoparticles have been widely used in the medical field. NPs have many advantages as drug carriers, such as variable shapes and sizes, stable interactions with ligands, high carrier capacities, and ease of binding to hydrophilic and hydrophobic drugs. In addition, the small volume and submicron size of NPs allows penetration of the cell tissue gap and easy cellular uptake. Furthermore, cells take up NPs mainly through endocytosis, which does, not damage the phospholipid bilayer cell membrane, and NPs easily pass through the smallest capillaries in the human body (Verma et al., 2008; Zanella et al., 2017). NPs can also be engineered for sustained and controlled drug release, and various biodegradable polymers (such as gelatin, chitosan, and biopolysaccharides), as well as synthetic macromolecules (such as polyethylene glycol (PEG), polylactic acid (PLA) and polylactic acid-polyglycolic acid copolymer (PLGA)), which are degraded *in vivo* mainly through hydrolysis of the carbon chain, can be used to construct a sustained-release system for drug delivery (Moreno Raja et al., 2019). Depending on the method of preparing the delivery system, the material and the loaded drug, the duration of drug release can be extended to hours or even months.

### 4.1 Nanoparticle carrier preparation method

To date, many methods have been developed for encapsulating therapeutic agents in NP carriers, and the choice of encapsulation method depends largely on the type of drug to be encapsulated and the clinical use. There are two main ways to prepare NP carriers: bottom-up and top-down approaches.

#### 4.1.1 Bottom-up approaches

Bottom-up approaches include two main synthesis methods, emulsion polymerization and dispersion/precipitation polymerization, through which many types of NPs can be prepared. First, monomers form a colloid or homogeneous solution in the presence of stabilizers in the liquid phase, and after a polymerization initiator is added under certain conditions, the reacting monomers will continue to undergo chemical polymerization reactions, and discrete nanospheres will be formed with the growth of the polymerization chain (Duan et al., 2010; Gao et al., 2021). The drug to be contained in the NPs is added to the solution prior to polymerization, allowing the drug to be encapsulated in the core of the NPs (Xiong et al., 2016). Although dispersed particles of manageable size are obtained using this method, residual reagents may be retained in the core of the NPs, presenting many limitations in biomedical applications.

#### 4.1.2 Top-down approaches

Top-down approaches are the most commonly used techniques for NP preparation and include three categories: oil-in-water (O/W) emulsions, water-in-oil-in-water (W/O/W) emulsions and nanoprecipitation. The emulsion method is mainly used to prepare NPs from materials such as PLA, PLGA, and polycaprolactone (PCL) since the emulsion method requires the polymer to be a volatile but water-insoluble solvent (such as dichloromethane) (Ito et al., 2010; Kanakubo et al., 2010). Nanoprecipitation is a relatively simple nanopreparative method and is one of the best methods for preparing PLGA. Since the nanoprecipitation method has a low encapsulation rate for hydrophilic drugs, it is mainly used to encapsulate hydrophobic drugs (Almoustafa et al., 2017).

### 4.2 Commonly used drug-carrying nanoparticles

#### 4.2.1 Mesoporous silica nanoparticles

**FIGURE 3:**
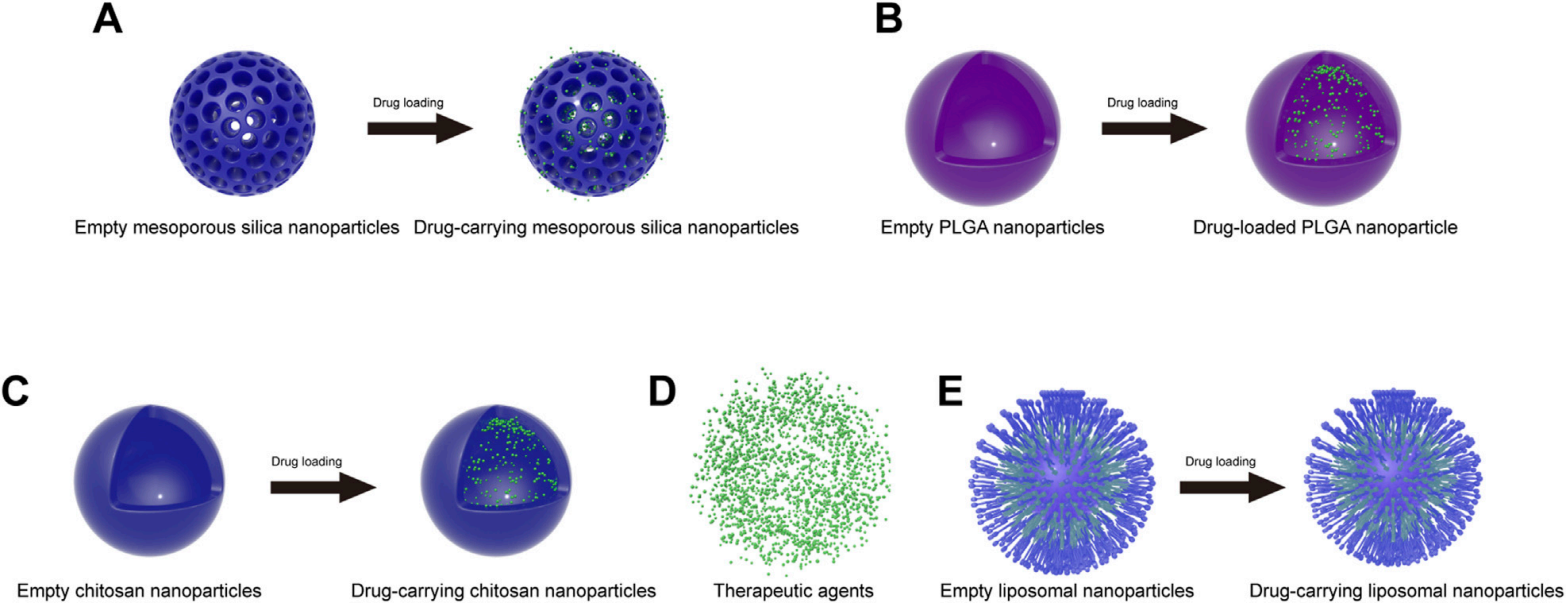
Schematic of commonly used nanoparticles before and after drug loading. **(A)** Mesoporous silica nanoparticles; **(B)** PLGA nanoparticles; **(C)** chitosan nanoparticles; **(D)** therapeutic agents; **(E)** liposome nanoparticles.

Recently, mesoporous silica nanoparticles (MSNPs) have been commonly used as carriers for *in vivo* drug delivery because of their low cost, low toxicity, slow drug release, high drug loading, biocompatibility, increased drug solubility, pore size tunability, easy surface functionalization and high specific surface area (Gupta et al., 2023) (Figure 3A). MSNPs are usually synthesized from silica sources, catalysts and surfactants by evaporation-induced self-assembly, electrochemically assisted sol-gel methods, quenching, ultrasound-assisted synthesis, and microwave-assisted synthesis (Gupta et al., 2023). The particle size, pore volume and shape of MSNPs can be controlled by altering the synthesis process. In addition, we can perform surface modification of MSNPs to improve their properties, such as targeting, slow release and biocompatibility, *in vivo* (Ahmed et al., 2022). Currently, MSNPs can achieve targeted drug delivery by regulating the pH response, redox response, enzyme response, thermal response, reactive oxygen response, magnetic response, ultrasonic response, light response, and electrical response (Gupta et al., 2023).

#### 4.2.2 Polylactic acid-polyglycolic acid copolymer nanoparticles

Polylactic acid–polyglycolic acid (PLGA) is a biodegradable copolymer. Due to its excellent biocompatibility, biodegradability, biosafety and multifunctionality, PLGA was approved by the U.S. Food and Drug Administration (FDA) for use in the biomedical field and is among the most commonly used polymers in the biomedical field (Pinto et al., 2022) (Figure 3B). PLGA is formed by the irregular polymerization of polylactic acid (PLA) and polyglycolide (PGA). PLA is an aliphatic polyester with biodegradable properties made by the direct condensation of lactic acid monomers. PLA is degraded by cells in the body after hydrolytic fracture to produce lactic acid, which is a natural waste product produced by the human body. Therefore, PLA is very biocompatible and thus suitable for biological applications. PGA is a crystalline hydrophilic polymer formed by the ring-opening polymerization of glycolide monomers and undergoes rapid degradation. The degradation product is glycolic acid, which is converted into glycine in the body and subsequently expelled from the body. PLGA combines the properties of PGA and PLA monomers. Since PGA and PLA have very different properties, it is possible to adjust the ratio and molecular weight of the copolymer (lactic acid/glycolic acid) to obtain PLGA copolymers with different properties (crystallinity, degradation time, etc.) (Moreno Raja et al., 2019).

Recently, PLGA nanoparticles have been used as drug carriers for various small and large molecules. Examples include cancer therapeutics (Li et al., 2023; Yadav et al., 2023), diabetes therapeutics (Pang et al., 2023; Sanapalli et al., 2023) and anti-inflammatory drugs (Aldayel et al., 2016). These compounds can even be used for gene therapy (Frede et al., 2016).

#### 4.2.3 Chitosan nanoparticles

Chitosan (CS) is a natural cationic copolymer composed mainly of randomly distributed N-acetylglucosamine and D-glucosamine (Cao et al., 2022b). This polysaccharide is mainly derived from crustacean shells by the alkaline deacetylation of chitin. Moreover, CS has excellent biocompatibility, nontoxicity, adhesion and biodegradability, as well as ease of modification. CS is broken down *in vivo* by lysozyme into oligosaccharides or individual monomers, which are easily metabolized by cells. Therefore, CS has received great attention in biological fields. In particular, CS is often used as a carrier for *in vivo* drug delivery (Figure 3C). Examples include cancer therapeutics (Chakraborty et al., 2023) and Alzheimer’s disease therapeutics (Yang et al., 2023). These therapeutic agents can be encapsulated inside the nanoparticles (Figure 3D). Currently, CS nanoparticles can be prepared by ionic gelation, complex gelation, coprecipitation, microemulsion, emulsified solvent diffusion and reverse micellization (Cao et al., 2022b).

#### 4.2.4 Liposomal nanoparticles

Liposomes are concentric bilayer spherical lipid vesicles that are formed mainly from natural or synthetic phospholipids and are structurally similar to cellular bilayer phospholipid membranes. Inside the liposome is an aqueous core, into which hydrophilic drug molecules can be loaded, while hydrophobic drugs can be encapsulated by the bilayer membrane (Wacker, 2013) (Figure 3E). Liposomal nanoparticles (LNPs) are often used as carriers for *in vivo* drug delivery due to their reduced drug toxicity, slowed release effect, improved drug stability, degradability and excellent biocompatibility. Currently, LNPs are mainly used to treat cancer (Graván et al., 2023). LNPs have also been studied for their use in the treatment of inflammatory and neurological disorders (Correia et al., 2022; Sung et al., 2022).

#### 4.2.5 Metallic nanoparticles

Gold nanoparticles (GNPs) are available as spheres, rods, shells and cages and can be used in different applications depending on their shape. Among these materials, gold nanospheres can be used as delivery carriers for drugs *in vivo*. GNPs ensure that drugs are delivered to designated locations in the body, which can lead to the release of drugs at targeted locations and increase penetration into cells, thus reducing the amount of drug needed and lowering the systemic toxicity of the drug.

Silver nanoparticles (SNPs) have the same shape as do GNPs and include spheres, rods and shells. SNPs have antibacterial and anti-inflammatory effects and can induce cell apoptosis and inhibit the secretion of tumor necrosis factor (TNF-α), thereby promoting the rapid healing of damaged areas. In addition, SNPs can be used as antiplatelet agents to prevent vascular restenosis (Loomba and Scarabelli, 2013).

In addition, metal nanoparticles of different sizes and materials also have an impact on the therapeutic effect. In the study of Amani et al. (2021), they used computers to simulate the adhesion surface density (SDP) of metal particles with different particle sizes (ranging from 400 to 1,000 nm) and materials (SiO_2_, Fe_3_O_4_, NiO_2_, silver, and gold) on atherosclerotic plaques. The research results showed that for large particles (800 and 1,000 nm), the amount of SDP on the plaque significantly increases with the increase of injected particle number, and the increase in SDP was about 50% higher than that of small particles (400 and 600 nm). The injection of medium density metal particles (Fe_3_O_4_, NiO_2_, silver) resulted in higher SDP. In addition, the research results also indicated that the affinity, geometric characteristics, and biophysical factors of nanoparticle adhesion exceeded the influence of particle density differences on SDP. Overall, this provides insights for the prevention of vascular restenosis using metal nanoparticles.

## 5 Advantages of nanoparticle-loaded drugs in preventing vascular restenosis after vascular injury

Currently, vascular restenosis is prevented by providing antiproliferative or anti-inflammatory agents through drug-eluting stents (DESs) (Habara et al., 2015; Ali et al., 2019; Lin et al., 2023; Wang et al., 2023). A DES is used to prevent vascular restenosis by applying antiproliferative or anti-inflammatory drugs to a bare metal stent with slow sustained release of the drug at a localized location. However, there are obvious drawbacks to preventing vascular restenosis by DESs, such as the limited amount of drug released and to the potential for uncontrolled drug release (Yao et al., 2021). In addition, these therapeutic agents inhibit EC proliferation and migration, which leads to prolonged healing at the site of endothelial injury and ultimately exacerbates vascular restenosis (Torii et al., 2020). Moreover, implanting foreign substances directly into the body can lead to inflammation and thrombosis, which ultimately results in a lack of therapeutic efficacy and even exacerbates vascular restenosis (Inoue et al., 2011). In addition, if exogenous agents for the treatment of vascular restenosis are delivered via systemic administration, these therapeutic agents are highly susceptible to phagocytosis and clearance by the mononuclear phagocyte system in the body, thus limiting the bioavailability of the therapeutic agents in the body. Therefore, there is an urgent need to find a nontoxic, slow release, targeted and biocompatible drug-loading carrier to prevent vascular restenosis.

**FIGURE 4:**
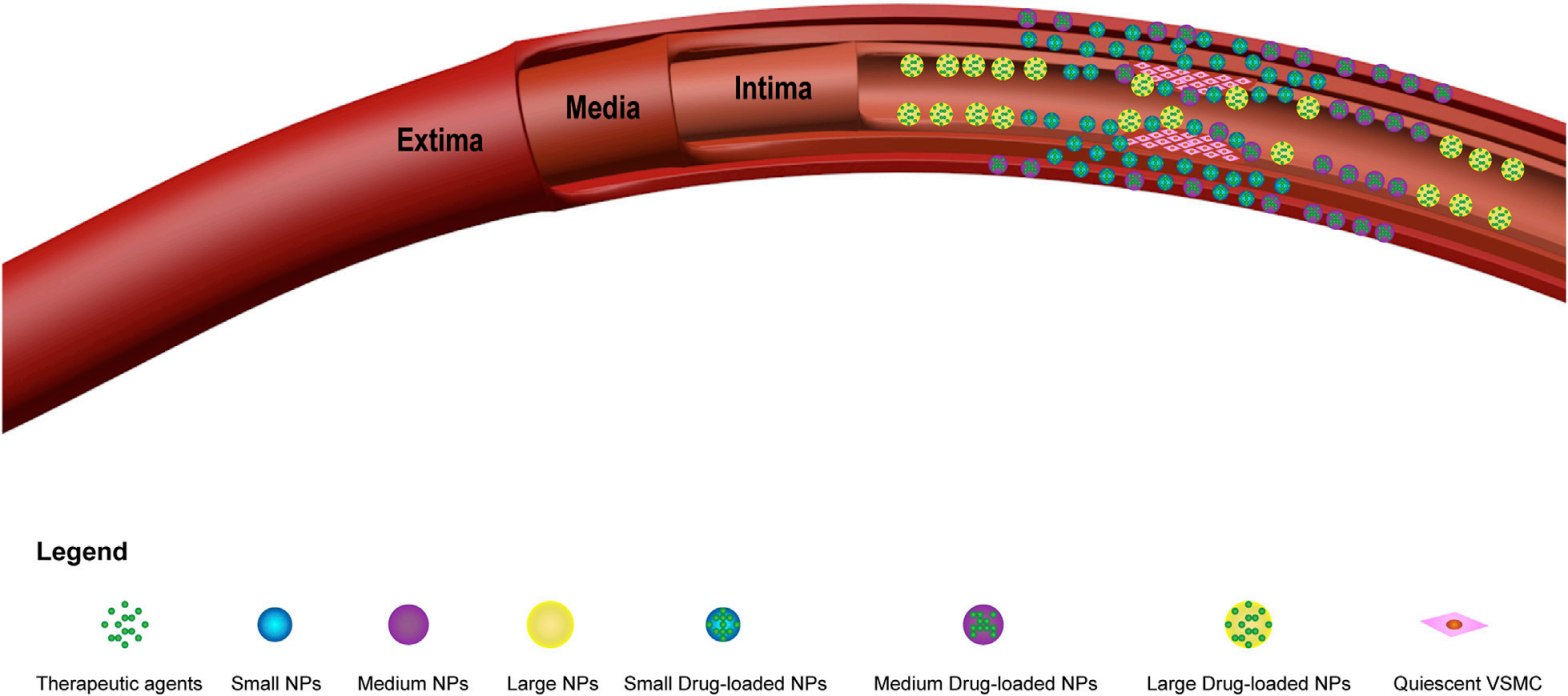
Diagram of different sizes of drug-carrying nanoparticles in vascular restenosis. The abbreviations used are as follows: small NPs (small nanoparticles); medium NPs (medium nanoparticles); large NPs (large nanoparticles); small drug-loaded NPs (small drug-carrying nanoparticles); medium drug-loaded NPs (medium drug-carrying nanoparticles); and large drug-loaded NPs (large drug-carrying nanoparticles). Small NPs (2–6 nm) are distributed throughout the vessel wall, medium NPs (93 nm) are distributed only within the tunica intima and external membrane of blood vessels, and large nanoparticles (150–450 nm) are found only within the tunica intima of blood vessels.

After intervention, most of the drugs are washed away by the blood flow, it is very difficult to achieve local drug delivery, and only a very small portion of the drugs act on the endothelial damaged area. Moreover, due to their submicron volume and excellent tissue penetration, nanoparticles greatly increase the probability of local administration. Westedt et al. (2002) showed that 100–200 nm NPs were deposited in the inner regions of the vessel walls, while 514 nm NPs mainly aggregated on the surface of vessel lumens. In addition, experiments by Rome et al. (1994) showed that the efficiency of drug delivery is related to the size of the NPs. In their experiments, the permeability of the vascular wall was investigated using horseradish peroxidase (HRP) (diameter: 2–6 nm), fluorescently labeled latex microspheres (diameter: 93 nm), and colloidal carbon (diameter approximately 150–450 nm) as tracer particles. The results showed that the delivery of small NPs (2–6 nm) was better than that of the other particles, as the NPs were distributed throughout the entire blood vessel wall. Medium-sized NPs (93 nm) were distributed only within the tunica intima and tunica adventitia of the vessels, while large NPs (150–450 nm) were present only in the tunica intima (Figure 4). Moreover, due to the ultramicroscopic nature of the nanoparticles, there was no obvious foreign body reaction when the cells ingested them.

Depending on the physicochemical properties of the therapeutic agent, the encapsulate therapeutic drug can constitute up to 30% w/w of the NPs (Liu et al., 2023b). In addition, by adjusting the preparation parameters of the NPs and the composition of the polymers, the NPs can be designed to decompose slowly at the site of vascular damage. The drugs inside the NPs are slowly released for a week to several months, thus achieving sustained inhibition of vascular restenosis. Akhlaghi et al. (2019) prepared LNPs loaded with curcumin (CUR) to inhibit vascular restenosis after angioplasty. During the *in vitro* release experiment of CUR, the optimized drug-loaded nanoparticles exhibited sustained and slow release at 37°C, and there was no burst of drug release at any point. The release time was extended to 21 days, during which 53.8% of the CUR was released, ensuring a controlled concentration of the drug at the site of endothelial injury. Furthermore, *in vivo* experimental results in mice showed that the use of CUR NPs for local delivery significantly inhibited the growth of the neointima and reduced the intima/media ratio. In addition, Westedt et al. (2007) prepared poly(vinyl alcohol)-grafted poly(lactide-co-glycolide) (PVA-g-PLGA) nanoparticle carriers loaded with paclitaxel to inhibit vascular restenosis. In an *in vitro* paclitaxel release assay, PVA-g-PLGA NP-loaded paclitaxel was shown to undergo a burst of drug release in the first 24 h, followed by a slow and continuous release of paclitaxel over a 22-day period. *In vivo*, the intimal area of vessels treated with NP-loaded paclitaxel was reduced by as much as 50% compared with that of control vessels, exhibiting excellent prevention of vascular restenosis.

NPs can also improve the water solubility of drugs. Currently, the water solubility of many drugs is relatively poor, and NPs encapsulation can improve their water solubility for better application to disease treatment. According to the results of Gou et al. (2021), carboxyl-functionalized mesoporous silica nanoparticles (MSN-COOH) were prepared for the controlled delivery of nonsteroidal anti-inflammatory drugs. Carboxylate functionalization significantly increased the dissolution of nimesulide (NMS) and indomethacin (IMC) compared to that achieved by unfunctionalized MSNPs. The *in vivo* biological effects showed that NMS and IMC were significantly more bioavailable after MSN-COOH encapsulation than after MSNP encapsulation and that MSN-COOH exerted stronger anti-inflammatory effects by delivering more NMS and IMC *in vivo*.

## 6 Nanoparticles for the prevention of vascular restenosis

### 6.1 Drug delivery

According to the mechanism of vascular restenosis, the drugs selected include anti-inflammatory drugs, such as curcumin (Akhlaghi et al., 2019), colchicine (Deftereos et al., 2013; Kong et al., 2015), and dexamethasone (Ji et al., 2022); antiproliferative agents, such as curcumin (Akhlaghi et al., 2019), colchicine (Deftereos et al., 2013; Kong et al., 2015), honokiol (Wei et al., 2020), paclitaxel (Chan et al., 2011; Cao et al., 2022a; Zhu et al., 2022), and sirolimus (rapamycin) (Feng et al., 2016; Haeri et al., 2017; Bai et al., 2022); drugs that promote endothelial regeneration, such as pitavastatin (Liu et al., 2017a); and anticoagulant drugs that counteract platelet aggregation and thrombus formation, such as heparin (Betala et al., 2020). Among these agents, curcumin and colchicine inhibit both the inflammatory response and cell proliferation.

#### 6.1.1 Polymer nanoparticle drug delivery

Polymer NPs have the advantages of biocompatibility, biodegradability, slow release and easy surface modification and are currently often used as carriers for drug delivery *in vivo*. The most prominent drug-carrying polymer NPs currently available for the prevention of vascular restenosis are poly(lactic-co-glycolic acid) (PLGA), polylactic acid (PLA)/polycaprolactone (PCL) (PLA/PCL) NPs and nanoaggregates. Among them, the most commonly used drug carriers for preventing vascular restenosis are PLGA NPs, which have slow release and a high encapsulation rate. In the experiments of Ji et al. (2022), PLGA NPs were prepared for the encapsulation and delivery of dexamethasone. The nanoparticle size range prepared in this experiment was 200–300 nm, with an average of 258 nm. *In vitro* drug release experiments, showed that dexamethasone was released slowly within 30 days, and the release rate gradually decreased with time. Moreover, the rate of dexamethasone encapsulation by PLGA nanoparticles was measured to be as high as 99.2%. This study showed that PLGA-encapsulated dexamethasone has better physicochemical properties than free dexamethasone and can be applied for the treatment of vascular restenosis.

In addition, PLGA nanoparticles are easily surface-modified. Westedt et al. (2007) prepared poly(vinyl alcohol)-grafted-poly(lactide-co-glycolide) (PVA-g-PLGA) NPs with paclitaxel for the prevention of vascular restenosis. In another study, Mungchan et al. (2022) prepared PLGA NPs specifically modified with glycoprotein Ib alpha chain (GPIbα) and human single-chain antibody variable fragment (HuscFv) for the prevention of vascular restenosis. In addition, the chain length of PLGA can be changed, and PVA can be modified to load different drugs. The above properties indicate that the PLGA NPs are promising drug carriers for the prevention of vascular restenosis.

PLA and PCL are biocompatible and easily surface-modified and are also used as drug carriers for the prevention of vascular restenosis. Das et al. (2000) prepared PEG-modified PLA/PCL NPs to encapsulate and deliver colchicine. The results proved that the PEG-modified NPs effectively extended the release time of the drug. Tamboli et al. (2013) prepared PLA-PCL-PEG-PCL-PLA NPs to encapsulate and deliver steroids. The results proved that the PLA-PCL-PEG-PCL-PLA NPs effectively extended the release time of the drug. In another study, Park and Yoo (2006) used PEG and PLA to form PEG-PLA-PEG triblock copolymer nanoaggregates and reported that the drug encapsulation rate significantly increased when shorter PEG chains were used. Similarly, the drug release profile can be adjusted by altering the chain length of PEG, which will be useful in future studies of PEG-modified PLGA.

#### 6.1.2 Inorganic nanoparticle drug delivery

MSNPs are drug-carrying NPs with advantages over PLGA nanoparticles. MSNPs are characterized by low cost, low toxicity, slow release, high drug loading, good biocompatibility, increased drug solubility, pore size tunability, easy surface functionalization and high specific surface area. At present, MSNPs are not commonly used for preventing vascular restenosis, but these advantages are expected to make them the next-generation of commonly used drug carriers for the prevention of vascular restenosis. Wei et al. (2020) used MSNPs to encapsulate and deliver honokiol (HNK). The results showed that the MSNPs were virtually nontoxic the cells, and the synthesized HNK-MSNPs enhanced the potency and cellular uptake of HNK. MSNPs not only reduced the toxicity of HNK but also delivered HNK to VSMCs rapidly and efficiently. In addition, HNK-MSNPs inhibited the proliferation and migration of VSMCs *in vitro*, and *in vivo* experiments also proved that HNK-MSNP administration significantly inhibited the proliferation of neointima.

#### 6.1.3 Liposomal nanoparticle drug delivery

Liposomes have the advantages of superior biodegradability and biocompatibility, reduced drug toxicity, a high drug loading rate and sustained drug release. Haeri et al. (2011) prepared liposomal nanoparticles (LNPs) loaded with sirolimus (SIR) with a loading rate of up to 98% and demonstrated that the NPs inhibited IH and prevented vascular restenosis. Later, Haeri et al. (2017) prepared LNPs modified with CS containing SIR for the prevention of vascular restenosis. In this study, it was demonstrated that encapsulating LNPs with chitosan improved their ability to prevent vascular restenosis. Ang et al. (2020) similarly prepared LNPs loaded with SIR, although they used exogenous injection to introduce the NPs into the site of the injured vascular external membrane. This mode of administration reduces the effect of the drug on reendothelialization and decreases the loss of therapeutic drug. In addition, Chen et al. reported that dimyristoyl phosphatidylcholine (DMPC) liposomal nanoparticles encapsulating HNK had the greatest inhibitory effect on the proliferation of VSMCs (Chen, 2009). In the study by DiPasquale et al. (2022), it was found that ceramides can stabilize the structure of liposomes to a certain extent. However, the shorter the length of the acyl chain of the lipid is, the more unstable the liposome will be. Therefore, we can change the properties of liposome-loaded drugs by changing the length of the acyl chains, which provides a new direction for the future design of drug-loaded liposomes.

Although LNPs are highly biocompatible, they are very likely to retain organic reagents during the preparation process, which decreases the overall biocompatibility of LNPs. Recently, Akhlaghi et al. (2019) prepared LNPs loaded with CUR using only triacylglycerol and lecithin without any organic reagents. The method was characterized as green and nontoxic. The NPs showed improved biocompatibility, strong stability, high drug loading capacity and good biodegradability. In addition, the optimized CUR LNPs had a smaller particle size and lower polymer dispersity index (PDI), which are conducive to intertissue permeation and cellular uptake.

#### 6.1.4 Nanoparticles that carry dual drugs

The use of NP carriers that carry only one therapeutic agent is likely to be insufficient because some therapeutic agents act only on one of the processes involved in restenosis. Drugs such as dexamethasone (Ji et al., 2022), paclitaxel (Zhu et al., 2022), and heparin (Li et al., 2017) can act only individually to stymie the inflammatory response, inhibit cell proliferation or counteract thrombus formation to inhibit the vascular restenosis. However, the formation process of vascular restenosis is very complex, mainly including platelet aggregation, proliferation and migration of vascular smooth muscle cells, intimal hyperplasia, and inflammatory response. Inhibiting a single process of vascular restenosis formation cannot completely prevent vascular restenosis, as it may occur through other processes. Therefore, NPs carrying dual therapeutic agents are better able to prevent vascular restenosis. Betala et al. (2020) prepared polylactic acid-hydroxyacetic acid-grafted polyethyleneimine (PgP) nanoparticles that can carry two drugs, sirolimus (SIR) and heparin (Hep). The results of this study showed that treatment with NPs carrying dual drugs not only reduced the migration and proliferation of VSMCs but also reduced collagen deposition and thickening of the neointimal region. However, these NPs cannot achieve targeted drug delivery. In addition, many synergistic combinations of hydrophilic and hydrophobic drugs are used to prevent vascular restenosis. To address this approach, Liu and He (2015) modified CS NPs to obtain a drug carrier that can release both hydrophilic and hydrophobic drugs, which has very good application prospects in the multidrug prevention of vascular restenosis.

### 6.2 Targeted drug delivery nanoparticles

Although drug-carrying NPs can effectively prevent vascular restenosis, they are administered locally and do not have targeting properties. When blood flow is restored to the damaged region of the blood vessel, some of the drug-carrying NPs will be washed away by the blood, which may result in a lack of therapeutic efficacy. To address this problem, some researchers have improved and prepared NPs for targeted drug delivery.

#### 6.2.1 Single-targeted nanoparticles

Targeted drug delivery by NPs has been widely used to treat cancer, and NPs have been developed for targeted drug delivery to prevent vascular restenosis. In a study by Chan et al. (2011), NPs encapsulating paclitaxel were prepared for the targeted delivery of paclitaxel to the damaged vascular basal layer. The nanoparticles were administered intravenously for systemic delivery, and the surface was modified with a collagen type IV targeting peptide, which primarily targets collagen IV in the vascular basement membrane (50% of the vascular basement membrane). In a rat carotid artery injury model, the targeted NPs reduced arterial stenosis by 50% compared to that in the control group. The NPs not only improved cellular tolerance to paclitaxel but also increased the duration of drug release and successfully targeted vascular restenosis, which may lead to effective targeted treatment of vascular restenosis.

In another study, Zhu et al. (2022) successfully constructed pH-responsive poly(lactic-co-glycolic acid) (PLGA) NPs (PTX-NaHCO_3_-PLGA NPs) loaded with paclitaxel (PTX) and NaHCO_3_. The NPs exhibited significant pH sensitivity and good biosafety *in vivo*. The main principle of this pH response is that the extracellular pH is 7.4 in a normal physiological state and is lower at the site of vascular inflammation, indicating that the tissue is acidic. Sodium bicarbonate reacts with hydrogen ions under acidic conditions to form salt and carbonic acid, which further decompose into carbon dioxide and water and are ultimately excreted by the body. To utilize this property, both sodium bicarbonate and PTX were encapsulated into PLGA. When the NP reaches the acidic location of vascular restenosis, the sodium bicarbonate inside the NP reacts with hydrogen ions to generate carbon dioxide gas, which cleaves the PLGA NP, thus achieving the targeted release of PTX at the site of vascular restenosis. According to the *in vitro* pH data, the PTX-NaHCO_3_-PLGA NPs effectively released PTX under acidic (pH = 6.0) conditions. No significant damage to major organs of the body was observed after the administration of PTX-NaHCO_3_-PLGA NPs, suggesting that the NPs are biologically safe. In in vivo experiments, PTX-NaHCO_3_-PLGA NPs were found to inhibit VSMC proliferation. Therefore, PTX-NaHCO_3_-PLGA NPs are good potential therapeutic agents for targeting vascular restenosis. However, the *in vivo* presence of these NPs may be limited by the fact that the surface of the NP is not modified: the NP is simply loaded with the drug and subsequently used to treat vascular restenosis, so the drug is likely to be released very quickly once it reaches the site of inflammation. Moreover, if inflammation is present in other parts of the body, the NPs will release the drug in other parts of the body as well.

In addition, Feng et al. prepared two types of targeted rapamycin (RAP)-loaded NPs to respond to the slightly acidic pH in the inflammatory microenvironment and to highly reactive oxygen species (ROS) (Feng et al., 2016). In this study, acetalized β-cyclodextrin (β-CD) materials (Ac-bCD) were used as pH-responsive nanomaterials, and ROS-responsive materials (Ox-bCD) were prepared from β-CD. Ac-bCD rapidly hydrolyzes and releases the drug under acidic (pH = 5.0) conditions, indicating its pH sensitivity. Ox-bCD rapidly hydrolyzes and releases the drug it carries in the presence of hydrogen peroxide, demonstrating its oxidative sensitivity. The main mechanism for targeting vascular restenosis in this study was endothelial damage after interventional procedures, which led to inflammation and in turn to a decrease in pH at the injury site. β-CD is an acid-sensitive material that allows pH targeting at the injury site. In addition, large amounts of ROS are generated at sites of vascular damage, so oxygen-sensitive materials can be used to achieve ROS targeting of restenosis sites in vessels. In an *in vitro* NP uptake assay in VSMCs, both pH- and ROS-responsive NPs exhibited improved release compared with untargeted NPs. In addition, compared with nonresponsive nanoparticles, pH- and ROS-responsive NPs both increased the activity of RAP, thus improving the antiproliferative effect of RAP on VSMCs.

In another study, Kona et al. (2012) modified PLGA NPs loaded with dexamethasone using the glycoprotein Ib alpha chain (GPIbα) of platelets. The specific binding properties of GPIbα to highly expressed P-selectin and von Willebrand factor (vWF) on the damaged endothelium are then utilized to achieve targeted vascular restenosis. These NPs were very effective *in vitro* and effectively targeted sites of vascular damage. However, how NPs bind to damaged sites in the presence of platelets is unclear since platelets also accumulate at damaged sites in blood vessels. Recently, cell membrane-encapsulated nanoparticles have been a topic of intense research interest. Wang et al. (2018) and Li et al. (2022) both used platelet membranes to encapsulate nanoparticles to achieve targeted prevention of vascular restenosis. NPs encapsulated by platelet membranes retain the properties of platelets and exhibit higher biocompatibility, improved targeting, faster biodegradability, and longer *in vivo* circulation than unencapsulated NPs.

Others have utilized physical methods for vascular site-targeted delivery. Polyak et al. (2016) utilized magnetism to deliver ECs enriched with superparamagnetic nanoparticles (MNPs) to the site of stent implantation *in vivo*. Although the results of this study showed that magnetically mediated targeting of ECs to the stent location is effective at preventing vascular restenosis, safety and feasibility assessments are still needed. Nevertheless, this study provides a novel targeting approach to prevent vascular restenosis.

#### 6.2.2 Dual-targeted nanoparticles

The use of single-targeted NPs for the prevention of vascular restenosis is a good method, but dual-targeted NPs have obvious advantages in the treatment of vascular restenosis. Dual-targeted NPs can reduce the off-target effects of NPs and facilitate the accurate delivery of drugs to locations prone to vascular injury. Mungchan et al. (2022) prepared PLGA NPs specifically modified with glycoprotein Ib alpha chain (GPIbα) and human single-chain antibody variable fragment (HuscFv), which could target the damaged subendothelial layer involved in vascular restenosis. Moreover, the NPs captured EPCs and accelerated endothelial regeneration. In addition, Wang et al. prepared dual-targeted NPs that can inhibit the proliferation and migration of VSMCs while promoting the proliferation and migration of ECs (Wang et al., 2021). The NPs were shown to inhibit the proliferation and migration of VSMCs while promoting the proliferation and migration of ECs in an *in vitro* cellular assay, but an *in vivo* assay was not performed in this study. The *in vivo* microenvironment of ECs and VSMCs is much more complex than that of simple culture medium *in vitro*. Further *in vivo* experiments are needed to verify the preventive effect of these NPs on vascular restenosis.

The environment of reduced pH and high ROS at the site of vascular injury can be specifically targeted. Previously, Feng et al. (2016) prepared NPs targeting either pH or high ROS concentration for this environment. Recently, in the studies of Zhang et al. (2020), PH/ROS dual-responsive NPs were prepared by adjusting the weight ratios of the PH-sensitive material ACD and the ROS-sensitive material OCD. Moreover, the NPs were modified with a peptide (KLWVLPKGGGC) targeting collagen type IV (Col-IV) to further improve their targeting.

Hao et al. (2022) prepared platelet membrane-encapsulated imidazole (IM)-modified methyl polyethylene glycol-polyaspartic acid (mPEG-PAsp-IM) pH-responsive PLGA NPs loaded with an endothelium-protective epigenetic inhibitor (JQ1). Blood vessel injury causes platelet aggregation, and NPs encapsulated in platelet membranes inherit the ability to aggregate at the site of vascular injury. After encapsulation in a platelet membrane, the nanoparticles exhibited higher biocompatibility, improved targeting and an extended circulation time *in vivo*. Moreover, after modification, the surface of the NPs had pH-targeting properties, and the NPs localized to sites of vascular restenosis. In addition, Liu et al. (2021) prepared macrophage membrane (MM)-coated ROS-responsive nanoparticles with encapsulated rapamycin (MM@PCM/RAP) for the treatment of IH. The research results indicated that MM@PCM/RAP has excellent biocompatibility and the ability to target sites of vascular injury. It can also effectively inhibit the pathological process of intimal injury, preventing intimal hyperplasia.

In the study by Shamloo and Forouzandehmehr (2019), the targeting ability of Sialyl Lewis^x^ (sLe^x^), P-selectin aptamer (PSA), and ICAM-1 antibody (abICAM) as surface targeting ligands for nanoparticles was simulated using a computer. They innovatively utilized computers to simulate the modification of ligands onto the surface of nanoparticles, and then targeted the endothelial inflammatory areas. The results indicated that the dual targeted nanoparticles with a particle size of 800 nm modified by 95.1% sLe^x^ and 4.9% PSA were the optimal modification conditions. This has profound implications for our design of targeted nanoparticles.

Although targeting can be an effective way to prevent vascular restenosis, if the particle size is not suitable, the distribution of drug-carrying NPs at the site of vascular injury will be uneven. A particle of suitable size has a more desirable biodistribution. Zhao et al., 2021b) prepared nanoclusters coated with an RVX-208 platelet membrane, which not only utilized encapsulation in the platelet membrane and a high concentration of ROS for dual targeting of vascular injury sites but also transformed into smaller nanoparticles at the targeted site. Thus, the nanoclusters more readily achieved distribution within the vascular wall, achieving better prevention of vascular restenosis.

### 6.3 Gene delivery

Localized delivery of genes can directly inhibit vascular restenosis via cellular pathways more rapidly than can localized delivery of drugs. Currently, the gene carriers used for preventing vascular restenosis mainly include polymer NPs, LNPs and biological NPs.

#### 6.3.1 Polymer nanoparticles as gene carriers

Polymer NPs are most commonly used to carry genes in genetically prevented vascular restenosis. For example, Yang et al. (2008) verified the role of PLGA NPs as gene carriers and found that PLGA NPs loaded with the antisense monocyte chemotactic protein-1 gene significantly reduced IH. Subsequently, Yang et al. (2011) used PLGA copolymer as a gene carrier to encapsulate antisense monocyte chemotactic protein-1 (A-MCP-1) and prepared A-MCP-1 NPs for local gene therapy for vascular restenosis. A-MCP-1 NPs express antisense MCP-1 mRNA and inhibit the expression of MCP-1 mRNA after local delivery through catheters to the location of interventional procedures. In addition, chitosan (CS) is commonly used as a gene carrier because of its high biocompatibility and biodegradability. Currently, CS is often used as a gene carrier in the treatment of cancer, but it has relatively few applications in treating vascular restenosis. However, some people do use CS to carry genes for the prevention of vascular restenosis. Xia et al. (2013) prepared CS NPs carrying anti-platelet-derived growth factor β (PDGF-β). These NPs mainly prevented vascular restenosis by inhibiting the expression of proliferating cell nuclear antigen (PCNA) and PDGF-β in VSMCs.

#### 6.3.2 Biological nanoparticles as gene carriers

Compared with polymer NPs, albumin NPs are more biocompatible, biodegradable, nonimmunogenic and noncytotoxic (Lei et al., 2021). Ji et al. (2014) used albumin NP-loaded genes to reduce IH in blood vessels. However, the most widely used gene-loaded NPs are LNPs, which have a phospholipid bilayer structure and excellent gene-carrying capabilities. Although gene-carrying LNPs are widely used in cancer treatment, their use in preventing vascular restenosis is limited.

### 6.4 Nanoparticles combined with stents to prevent vascular restenosis

Currently, vascular restenosis is prevented by DESs supplemented with antiproliferative or anti-inflammatory agents, such as paclitaxel, everolimus, and rapamycin, which inhibit the migration and proliferation of VSMCs. However, DESs often cause late thrombosis, and the carried drug may inhibit EC growth, leading to delayed endothelial healing. Combining drug-carrying NPs with stents to prevent vascular restenosis is a very promising strategy. Yang et al. (2013) coated PLGA NPs with VEGF encapsulated in the outer shell and PTX loaded in the inner shell onto stents. The experimental results showed that the stent could sequentially release VEGF and PTX, inhibiting VSMC proliferation while promoting endothelial reendothelialization. However, the hydrophobicity of the PLGA surface may cause adverse reactions in vascular restenosis. Jc Bose et al. (2016) modified PLGA NPs via lipidation and prepared lipid-hybridized polymer NPs loaded with the antiproliferative drugs sirolimus (SIR) and propolis, respectively. *In vitro*, the NPs not only significantly inhibited the proliferation of human aortic smooth muscle cells (HASMCs) but also preserved the viability of human aortic endothelial progenitor cells (HAECs). Although no animal studies were performed, *in vitro* experiments showed that the NPs have potential for use in DESs.

Improving the biocompatibility of stents is a potential strategy for preventing vascular restenosis. In a study by Li et al. (2017), poly-L-lysine (PLL) NPs coated with heparin (Hep) were coated onto the stents. The experimental results showed that stents coated with heparin-loaded PLL (Hep/PLL) NPs promoted intimal reendothelialization, exerted anticoagulant effects, and had a more biocompatible stent surface. In another study (Liu et al., 2017b), the loading of laminin into Hep/PLL NPs further enhanced the biocompatibility of the stent surface. Moreover, Hep/PLL NPs loaded with laminin exhibited excellent prevention of platelet adhesion and thrombosis. In addition, the NP-modified stents promoted the proliferation of EPCs and ECs and the synthesis of NO.

Metal particles released from drug-eluting stents are one of the main causes of vascular restenosis. In the study of Xu et al. (2017), the use of TiO_2_ NPs to coat bare metal stents effectively reduced the release of nickel ions from the metal stents. Later, in the work of Cherian et al. (2022), cobalt-chromium (CC) stents were modified with TiO_2_ NPs alone, and intimal reendothelialization was promoted without either drugs or polymers.

The drug-eluting stents that are commonly used today are metal stents, but stents prepared with biodegradable materials offer advantages over metal stents. For example, Zhao et al. (2018) prepared SIR-loaded polymeric poly(DL-lactide) (PDLLA) NPs coated on biodegradable poly(L-lactide) (PLLA) stents. The experimental results showed that the inhibitory effect of the stent on VSMC proliferation was significantly greater than that on EC proliferation. This stent and the drug-carrying NPs used to prevent vascular restenosis are both biodegradable. This finding shows the potential of the use of stents for preventing vascular restenosis.

A summary of NP applications in the prevention of vascular restenosis is shown in Supplementary Table S1.

## 7 Improving drug-carrying nanoparticles

Although NP carriers have the advantages of sustained release, high drug loading, biocompatibility, targeting, and easy cellular uptake, individual nanoparticles must be surface-modified before they can act on the corresponding tissues and cells. Moreover, the clinical application of NPs is limited by their rapid clearance by the reticuloendothelial system (RES) *in vivo*. The following section summarizes improvements in NPs for slow drug release, extended circulation time *in vivo*, and targeted drug delivery.

### 7.1 Extended drug release time

NPs can increase the duration of drug release. Zhang et al. (2021) prepared MSNPs with different pore sizes to encapsulate eugenol. The results showed that the smaller the pore size of MSNPs was, the slower the release of eugenol was. This indicated that the release time of the drug could be extended by preparing MSNPs with smaller pore sizes, but it is also important to note that the concentration of the drug that can treat the disease must be reached while slowing the release. In addition, Chen et al. (2016) prepared PLGA nanoparticles modified with a biotin chitosan surface (Bio-CS-PLGA NPs) for encapsulating the anticancer drug epirubicin (EPB). The results of *in vitro* drug release experiments showed that the Bio-CS-PLGA NPs, CS-PLGA NPs, and PLGA NPs released the drug EPB slowly and continuously after 48 h, and 78%–84% of the EPB was released slowly within 10 days. Moreover, compared with unmodified PLGA nanoparticles encapsulated with EPB, bio-CS-modified PLGA nanoparticles encapsulated with EPB had a significantly lower drug burst release in the first 24 h. This finding suggested that this modification can regulate the concentration of drugs released by PLGA NPs, effectively preventing the rapid release of most drugs from PLGA within the first 24 h.

### 7.2 Extended circulation time of drugs in the body

#### 7.2.1 Polyethylene glycolization

Polyethylene glycol (PEG) is a nontoxic, electrically neutral, hydrophilic and highly biocompatible material that has been deemed safe by the U.S. FDA (Nascimento et al., 2018). PEG is generally modified onto the surface of NPs to form a hydrophilic shell, which prolongs the blood circulation time of NPs (Zalba et al., 2022). Abdel-Hameed et al. (2022) prepared PEGylated silver NPs (PEG/AgNPs) for the encapsulation and delivery of epirubicin. *In vivo* distribution experiments showed that PEG-modified NPs had reduced RES clearance and increased *in vivo* circulation time. In addition, the density of PEG on the surface of NPs is an important factor influencing their clearance behavior *in vivo*. For the same size of NPs, greater PEGylation reduces the clearance of NPs by RES until the density threshold is reached. Increasing the amount of PEG on the surface of the NPs provides little benefit when the density threshold is exceeded (Bertrand et al., 2017).

#### 7.2.2 Bionic membrane wrapping

Exogenous NPs are subject to RES limitations after they enter the body, even after PEGylation. In recent years, biomimetic nanomaterials, especially cell membrane-disguised NPs, have shown great potential in drug delivery due to their good biocompatibility, long blood circulation ability and specific lesion targeting ability (Liu et al., 2022; Liu et al., 2023a). Direct modification of NPs using natural biomaterials can simplify the fabrication drug-carrying NPs and improve the biocompatibility of the NPs, which greatly extends the circulation time in the body (Rampado et al., 2022).

#### 7.2.3 Changing the structure of nanoparticles

After exogenous nanoparticles enter the body, they will be cleared by the RES system. In recent years, a simple method that does not require surface modification of nanoparticles has been discovered. We can achieve the effect of prolonging the circulation time of nanoparticles *in vivo* by simply changing the particle size and structure of nanoparticles (Forouzandehmehr and Shamloo, 2018). The research results of Forouzandehmehr and Shamloo (2018) showed that appropriately increasing the size of nanoparticles and changing their sphericity can increase the adhesion effect of nanoparticles at the thrombus site, thereby achieving the effect of prolonging the circulation time of nanoparticles in the body.

### 7.3 Improved targeting capabilities

#### 7.3.1 Enhanced targeting by the ligand–receptor system

By utilizing ligand receptor specific binding, the NP surface can be modified by ligands with lesion site-specific targeting to achieve targeting of the lesion site. Based on the physiological and pathological characteristics of vascular restenosis, damaged ECs, proliferative and migratory VSMCs, and inflammatory cells can be targeted. In addition, the selection of targeted ligands requires consideration of the changes in small-molecule components during vascular restenosis. It is affected by different stages of vascular restenosis, including the acute, neutral, and chronic stages. For example, Wang et al. (2021) designed dual-targeted NPs combined with physical mixing to target ECs and VSMCs. The massive recruitment of inflammatory cells is one of the key features of vascular restenosis. Zhu et al. (2022) successfully prepared pH-responsive PLGA NPs loaded with PTX and NaHCO_3_. The NPs can mainly target the site of the inflammatory response involved in vascular restenosis.

#### 7.3.2 Bionic membrane wrapping

Platelets are derived from megakaryocytes produced by bone marrow stem cells and play important roles in hemostasis, coagulation, vasoconstriction and inflammation. After interventional surgery, tearing of the endothelium can occur, resulting in inflammation and platelet aggregation to coagulate the wound area and provide hemostasis (Jin et al., 2018). Inspired by this, after the encapsulation of NPs by platelet membranes, which have platelet properties, platelet-encapsulated NPs aggregate at the site of intimal injury in the same way as platelets, thus targeting vascular restenosis. Li et al. (2022) prepared platelet membrane-modified loaded interleukin 10 (IL10) nanoparticles (IL10-PNPs) to target macrophages. In this study, IL10 was used as the therapeutic agent, PLGA NPs were used as the delivery vehicle, and platelet membranes were used to wrap the surface of the PLGA NPs by ultrasound. The results of *in vivo* experiments showed that PLGA NPs modified with a platelet membrane carrying IL10 can aggregate at the site of vascular injury and promote the polarization of macrophages toward the M2 phenotype, thereby inhibiting inflammation and promoting tissue repair. In addition, the observation and analysis of vascular tissue sections showed that IL10-PNPs could effectively inhibit intimal hyperplasia, promote the repair of endothelial cells, and reverse the phenotypic transformation of VSMCs. In addition, Hao et al. (2022) prepared mPEG-PAsp-IM pH-responsive PLGA nanoparticles loaded with JQ1 and encapsulated them in a platelet membrane (PM). The experimental results showed that the NPs significantly inhibited intimal hyperplasia.

Erythrocyte membranes have also been used for NP modification due to their accessibility, biocompatibility and long *in vivo* circulation time. However, according to the current literature search, no researchers have used erythrocyte membranes for the treatment of vascular restenosis. This may be due to the lack of a mechanism for targeting vascular restenosis; however, in the future, surface modification of the erythrocyte membrane could be used to target vascular restenosis, and owing to the properties of the erythrocyte membrane itself, the modification of NPs has very good application prospects.

### 7.4 Improvement of gene transfection capacity

Gene transfection efficiency refers to the degree to which foreign DNA or RNA molecules are successfully introduced into target cells through exogenous means in a cell culture system, and are accepted and expressed by the target cells. A high transfection efficiency means that more target cells successfully receive the delivery and expression of exogenous genes. The gene transfection efficiency of NPs loaded with genes is very important for the prevention of vascular restenosis. In a study by Wang et al. (2019), the same polymer modified with PEG was found to have better transfection ability and lower cytotoxicity. Therefore, it was hypothesized that modifying other NPs with PEG could improve the transfection ability. However, since different NPs have different properties, it is impossible to ensure that PEG modification will definitely improve the transfection ability, and additional experiments are needed to verify this supposition. Additionally, transfection can be enhanced by using neutral nanoliposome-loaded genes (Ozpolat et al., 2014). Anchoring low-generation dendrimers to NPs may be another method, although no studies have indicated that this approach can improve transfection ability. However, some studies have shown that MSNPs coupled with lower-generation dendritic molecules can effectively deliver DNA to cells that are difficult to transfect (Wang et al., 2017).

### 7.5 Modelling for drug carrier design

Due to the nanoscale size of nanoparticles, complex vascular biochemical environment, dynamic and nonlinear delivery processes, drug metabolism and clearance, and extravasation between different barriers, targeted nanoparticle analysis and measurement *in vivo* are very difficult (Nichols et al., 2017). In recent years, computer designed models have enabled us to study, design, and predict treatment strategies based on nanomedicine delivery systems with less time and cost. In addition, the optimization and development of computational methods and models have created broad and practical insights for specific drug design and treatment interventions (Forouzandehmehr et al., 2022). When designing targeted nanoparticles for vascular restenosis, we can pre modify the surface of the nanoparticles on the computer according to the condition of the restenosed vessel to achieve the effect of targeting vascular restenosis. Moreover, we can design multiple targeted nanoparticles to compare their targeting effects and find the best targeted nanoparticles. Finally, the best targeted nanoparticles will be used for *in vivo* validation. This computer design model can greatly save us time and cost in designing targeted drugs.

## 8 Conclusions and prospects

This paper reviews nanocarrier strategies developed in recent years for the treatment of vascular restenosis, including MSNP carriers, PLGA nanoparticle carriers, LNP carriers, chitosan NP carriers, and NP carriers with surface modifications such as PEG, IM, and biomimetic membranes. The advantages of using NP carriers to treat vascular restenosis and how to improve the shortcomings of NPs are also described. Therapy for vascular restenosis via platelet membrane-encapsulated NPs has recently become a research hotspot, and we believe that this NP delivery strategy is very promising for the treatment of vascular restenosis. With a better understanding of the mechanisms of vascular restenosis, multifunctional NPs will be developed for treatment. In addition, higher drug loading, stronger targeting and longer *in vivo* circulation times can be achieved by different modifications of NPs. In the future, more effective NP delivery strategies will involve NP material selection, surface modification, and the structural design of multiple NPs. We hope that this review will inform the development of novel NP delivery strategies for the prevention of vascular restenosis.
