# Supplementary material for: Nanoparticle-based approaches for treating restenosis after vascular injury
Source: Front Pharmacol. 2024 Oct 24;15:1427651. doi: 10.3389/fphar.2024.1427651 (PMC11540800; doi:10.3389/fphar.2024.1427651)
Supplement: Supplementary file 1 [file Table1.docx]

**Supplementary Table 1** Nanoparticles in vascular restenosis

| Therapeutic agent | Nanodelivery system | Animal | Animal model | Application advantages | Findings | Reference |
| --- | --- | --- | --- | --- | --- | --- |
| Curcumin (CUR) | CUR loaded lipid-based nanoparticles | Male Sprague‒Dawley rats (350~400 g) | Rat carotid artery balloon injury | 1. No organic reagents in the preparation process; 2. High biocompatibility; 3. High blood compatibility; 4. Controlled drug release; 5. Suitable particle size | The intima/media ratio was significantly lower in the group of animals receiving CUR nanoparticles than in the control group, and VSMC proliferation and intimal hyperplasia were significantly inhibited. | (Akhlaghi et al., 2019) |
| Honokiol (HNK) | HNK encapsulated in mesoporous silica nanoparticles (MSNPs) | Male Sprague‒Dawley rats (450~550 g) | Rat carotid artery balloon injury | 1. High surface area; 2. Extended release; 3. Prevention of the recrystallization of therapeutic agents; 4. Excellent biodegradability and biocompatibility; 5. Reduced drug toxicity | MSNPs encapsulated with HNK significantly inhibited the proliferation and migration of VSMCs, better inhibited endothelial thickening, and reduced the toxicity of HNK. | (Wei et al., 2020) |
| Paclitaxel (PTX) | DSPE–PEG–peptide shell; PLGA core encapsulating PTX | Male Sprague‒Dawley rats (450 g) | Rat carotid artery balloon injury | 1. Systemic drug delivery; 2. Reduced drug toxicity; 3. Excellent biodegradability; 4. Effective targeting; 5. Extended sustained release time | The targeted NP group not only significantly reduced intimal hyperplasia but also improved PTX tolerance compared with saline, paclitaxel, or nontargeted NP control groups. | (Chan et al., 2011) |
| Dexamethasone (DEX) | PLGA NP-encapsulated DEX | Stenotic arteries of the lower calf in diabetic patients | —— | 1. Sustained release; 2. Combination with angioplasty treatment; 3. High encapsulation rate | Restenosis of lower limb vessels was suppressed in diabetic patients treated with DEX NPs compared with the control group. | (Ji et al., 2022) |
| Paclitaxel (PTX) | Poly(vinyl alcohol)-graft-poly(lactide-co-glycolide) (PVA-g-PLGA) with encapsulated PTX | Male New Zealand white rabbits (2.5~3 kg) | Rabbit both common iliac arteries balloon injury | 1. Nontoxicity; 2. Excellent biodegradability and biocompatibility; 3. Sustained release | Compared with the control group, PTX-loaded PVA-g-PLGA treatment reduced the intimal area of damaged vascular segments by 50%. | (Westedt et al., 2007) |
| Paclitaxel (PTX) | pH-responsive PLGA nanoparticles loaded with PTX and NaHCO_3_ (PTX-NaHCO_3_-PLGA NPs) | Male Sprague‒Dawley rats (270~320 g) | Rat carotid artery balloon injury | 1. Systemic drug delivery; 2. Good targeting; 3. Excellent biodegradability and biocompatibility | In vitro experiments demonstrated that PTX-NaHCO_3_-PLGA NPs had excellent PH responsiveness and biosafety by decomposing and releasing the drug under microacidic environments. In vivo experiments revealed that PTX-NaHCO_3_-PLGA NPs significantly inhibited intimal hyperplasia, VSMCs proliferation and migration. | (Zhu et al., 2022) |
| Rapamycin (RAP) | Rapamycin encapsulated by Ac-bCD or Ox-bCD NPs with lecithin and DSPE-PEG modification | Male Sprague‒Dawley rats (480~500 g) | Rat carotid artery balloon injury | 1. Systemic drug delivery; 2. Effective targeting; 3. Nontoxicity; 4. Easy surface modification; 5. Excellent biocompatibility | Both Ac-bCD and Ox-bCD can localize to damaged blood vessels within a short time and have excellent targeting properties. In vivo experiments demonstrated that Ac-bCD or Ox-bCD NP-loaded RAP can significantly inhibit the proliferation of VSMCs and intimal hyperplasia, and the effect of Ox-bCD NP-loaded drug is more obvious. | (Feng et al., 2016) |
| Antisense monocyte chemotactic protein-1 (A-MCP-1) | PLGA NPs loaded with A-MCP-1 plasmid | Male New Zealand White rabbits (3.5~4.0 kg) | Rabbit carotid artery balloon injury | 1. Gene delivery; 2. Protection of therapeutic agents from decomposition; 3. Excellent biocompatibility and biodegradability | In vivo experiments demonstrated that PLGA NPs could effectively deliver A-MCP-1 into the vessel wall. The intima/media ratio was effectively reduced. | (Yang et al., 2011) |
| Sirolimus (SIR) and heparin (Hep) | Poly(lactide-co-glycolide)-graft-polyethylenimine (PgP) loaded with SIR and Hep (SR-PgP-Hep) | In vitro: Fresh porcine hearts (6~8 months) | Porcine coronary artery balloon injury | 1. Double drug loading; 2. Improved water solubility of drugs; 3. High biocompatibility | SR-PgP-Hep NPs not only reduced the proliferation and migration of VSMCs, collagen deposition and intimal hyperplasia but also increased vascular permeability in vitro. | (Betala et al., 2020) |
| —— | PLGA NPs modified with glycoprotein Ib alpha chain (GPIbα) and human single-chain antibody variable fragment (HuscFv) (GPIbα-HuscFv-PLGA) | —— | —— | 1. Dual targeting; 2. Nontoxicity; 3. Favorable biocompatibility and biodegradability | GPIbα-HuscFv-PLGA NPs not only aggregated on the damaged subendothelial surface to inhibit platelet aggregation but also captured endothelial progenitor cells (EPCs) to accelerate vascular repair. | (Mungchan et al., 2022) |
| Endothelium-protective epigenetic inhibitor (JQ1) | Platelet membrane encapsulated and imidazole (IM)-modified methoxyl poly(ethylene glycol)-poly(aspartic acid) (mPEG-PAsp-IM) NPs loaded with JQ1 | Male Kunming mice (30~35 g) | Mouse carotid artery metal guide wire injury | 1. Platelet targeting; 2. Favorable biocompatibility; 3. pH targeting | The mPEG-PAsp-IM NPs loaded with JQ1 had multiple effects such as inhibiting inflammation, intimal hyperplasia, and the proliferation and migration of VSMCs and protecting endothelial cells. They also exhibited higher biocompatibility, the ability to target vascular injuries, and prolonged in vivo circulation after encapsulation in platelet membranes. | (Hao et al., 2022) |
| Interleukin 10 (IL10) | Platelet membrane-encapsulated PLGA NPs loaded with IL10 (IL10-PNPs) | Male Sprague‒Dawley rats (200~250 g; 8~12 weeks) | Rat carotid artery balloon injury | 1. Platelet targeting; 2. Long in vivo circulation; 3. Favorable biocompatibility and biodegradability | IL10-PNP targeted the site of vascular injury after wrapping in the platelet membrane. It inhibited the proliferation and migration of VSMCs and promoted vascular reendothelialization by targeting and modulating M2-type macrophage polarization. | (Li et al., 2022) |
| VEGF plasmid (pVEGF);  ERK2 siRNA (si-ERK2) | Dual-targeted nanoparticles prepared by physically mixing Arg-Glu-Asp-Val (REDV) and citraconic anhydride comodified ε-polylysine-encapsulated pVEGF (REDV-PP/VEGF) and Val-Ala-Pro-Gly (VAPG) and citraconic anhydride comodified ε-polylysine-encapsulated si-ERK2 (VAPG-PP/si-ERK2) | —— | —— | 1. Precise regulability; 2. Dual targeting; 3. Excellent biocompatibility and cytocompatibility | By physically mixing two NPs targeting endothelial cells (REDV-PP/VEGF) and targeting vascular smooth muscle cells (VAPG-PP/si-ERK2), it promoted endothelial cell proliferation and migration while inhibiting VSMC proliferation and migration. | (Wang et al., 2021) |
| Sirolimus (SIR) | CS-modified LNPs loaded with SIR | Male Sprague‒Dawley rats (350~410 g) | Rat carotid artery balloon injury | 1. Favorable biocompatibility; 2. Nontoxicity; 3. Sustained release | CS-modified LNP-loaded drugs can prevent vascular restenosis more effectively than conventional LNP-loaded drugs. | (Haeri et al., 2017) |
| Sirolimus (SIR) | LNPs containing SIR | Male Sprague‒Dawley rats (350~400 g) | Rat carotid artery balloon injury | 1. Favorable biocompatibility; 2. Nontoxicity; 3. Sustained release | LNP-loaded drugs inhibited intimal hyperplasia and prevented vascular restenosis. | (Haeri et al., 2011) |
| Sirolimus (SIR) | SIR-loaded polymeric micelle and PEGylated nanoliposome | Male Sprague‒Dawley rats (350~400 g) | Rat carotid artery balloon injury | 1. Favorable biocompatibility; 2. Sustained release; 3. Excellent biodegradability | Both phospholipid-based micelles and polyethylene glycolized liposomes can be used to carry drugs to prevent vascular restenosis, and phospholipid micelle drug carriers achieved better prevention of vascular restenosis. | (Haeri et al., 2013) |
| Sodium nitrite | LNPs loaded with sodium nitrite added to constructed scaffolds | —— | —— | 1. Nontoxicity; 2. Sustained release; 3. Favorable biocompatibility | Stents containing LNP-loaded drugs were more biocompatible and have better prevention of vascular restenosis than stents containing drugs alone. | (Amoabediny et al., 2023) |
| —— | Titania nanotextured cobalt-chromium (CC) stents | New Zealand white rabbits (2.5~3.5 kg) | Rabbit iliac artery stent implantation | 1. Rapid re-endothelialization of the endothelium; 2. No use of drugs; 3. High hemocompatibility | After surface modification with titanium dioxide nanoparticles, the stent did not require any drug or polymer modification and to exert antithrombotic and anti-restenotic functions and promote vascular reendothelialization. | (Cherian et al., 2022) |
| Rapamycin (RAP); Bivalirudin (BVLD) | Epigallocatechin gallate (EGCG) Cu NPs coated with RAP and BVLD (EGCG-Cu@ RAP/BVLD) | New Zealand white rabbits (~2.7 kg) | Rabbit abdominal aortae stent implantation | 1. Sustained release; 2. Reduce drug toxicity; 3. Promotion of vascular reendothelialization; 4. Favorable biocompatibility | Stents coated with EGCG-Cu@ RAP/BVLD NPs can promote endothelial healing, block thrombosis, modulate inflammatory responses and prevent vascular restenosis. | (Zhang et al., 2022) |
| Quercetin | Quercetin covalently attached to PLGA | —— | —— | 1. Sustained release due to covalent linkage; 2. No burst release | The covalently attached quercetin PLGA NP delivery system had a longer release time and better anti-VSMCs proliferation effect than the directly loaded PLGA NPs. | (Craciun et al., 2022) |
| Rapamycin (RAP) | Macrophage membrane-encapsulated ROS-responsive NPs loaded with RAP (MM @ PCM/ RAP) | Male C57BL/6 mice (20~22 g, 7 weeks) | Mouse carotid artery metal guide wire injury | 1. Selective targeting of inflammation; 2. Long in vivo circulation; 3. Sustained release; 4. Improved drug solubility; 5. Favorable biocompatibility; 6. ROS targeting | MM @ PCM/RAP nanoparticles can target inflammation sites, achieve targeted release at sites of high reactive oxygen species (ROS), inhibit VSMCs proliferation and migration and reduce intimal hyperplasia. | (Liu et al., 2021) |
| Paclitaxel (PTX) | PLGA NPs loaded with PTX via didodecyl dimethyl ammonium bromide (DMAB) surface modification | Male New Zealand rabbits (2.0∼2.5 kg, 8~10 months) | Rabbit abdominal aortic balloon injury | 1. Favorable biocompatibility; 2. Strong surface adsorption; 3. Excellent Biodegradability; 4. Sustained release | PLGA drug-carrying NPs can be stably adsorbed on the damaged endothelium and even penetrate the cells to release drugs to prevent vascular restenosis. | (Zhao et al., 2021a) |
| Sirolimus (SIR) | LNPs containing SIR (SIR-LIP NPs) | Female mixed breed swine (40~50 kg) | Porcine femoral artery balloon injury | 1. Improvement of drug stability; 2. Administration by exogenous infusion; 3. Reduction in the inhibitory effect of drugs on intimal reendothelialization; 4. Reduction in drug use | The use of exogenously injected drug-carrying NPs is a novel delivery modality to prevent vascular restenosis. SIR-LIP NPs inhibited the proliferation of VSMCs and reduced the inflammatory response. | (Ang et al., 2020) |
| —— | Immobilization of nano-Cu-MOFs by polydopamine coating | Male Sprague‒Dawley rats (approximately 300 g); Male New Zealand white rabbits (approximately 3 kg) | Rat abdominal aorta wire implantation;  Rabbit iliac artery stent implantation | 1. Favorable biocompatibility; 2. Long-term action; 3. Convenient modification of stent surface | The stent modified with Nano Cu-MOFs showed desirable NO release and copper ion delivery capabilities in vivo, and it had anticoagulant properties, promoted intima reendothelialization, and inhibited intimal hyperplasia. | (Fan et al., 2019) |
| Sirolimus (SIR) | SIR-loaded polymer poly(DL-lactide) (SIR-PDLLA) NPs | —— | —— | 1. Excellent biodegradability; 2. High biocompatibility; 3. Slow release | SIR-PDLLA NPs can be successfully coated onto the stents with sustained drug release properties. In vitro experiments showed that SIR-PDLLA nanoparticles not only effectively inhibited the proliferation of VSMCs but also did not inhibit the proliferation of endothelial cells. | (Zhao et al., 2018) |
| 2-(3,4-Dimethoxyphenyl)-3-phenyl-4H-pyrido[1,2-a] pyrimidin-4-one (DB103) | DB103-loaded cyclodextrin-based nanosponges (DB103-NSs) | —— | —— | 1. Favorable biocompatibility; 2. Nontoxicity; 3. Slow release; 4. Nonhemolytic behavior; 5. Easy sterilization | DB103-NS allowed a gradual release of drug with no significant initial drug burst. | (Coviello et al., 2017) |
| Heparin (Hep) | PLL mixed with Hep to construct Hep/PLL microspheres | —— | —— | 1. Improved cytocompatibility; 2. Good anticoagulation; 3. Promotion of intima reendothelialization | Hep/PLL microspheres promoted intima reendothelialization and improved anticoagulation. | (Li et al., 2017) |
| Laminin;  Heparin (Hep) | Heparin/poly-L-lysine NPs loaded with laminin | —— | —— | 1. Good anti-coagulation; 2. Better stability; 3. Sustained release | In vitro experiments demonstrated that Hep/PLL NPs loaded with laminin promoted the proliferation of EPCs and ECs as well as NO synthesis. | (Liu et al., 2017b) |
| REDV | Nano-TiO_2_ and REDV applied to the stent | Male New Zealand rabbits (2.5~3.0 kg) | Rabbit iliac artery stent implantation | 1. Good blood compatibility; 2. Better stability; 3. High biocompatibility | Metal stent modified with titanium dioxide nanoparticles and REDV reduced nickel ion release and promoted intima reendothelialization. | (Xu et al., 2017) |
| Endothelial cells (ECs) | Polylactide-based magnetic NPs | Male Lewis rats (350~400 g) | Rat carotid artery stent implantation | 1. Adoption of magnetically mediated targeting; 2. Favorable biosafety | Targeting endothelial cells rich in superparamagnetic nanoparticles (MNPs) to the site of stent implantation in vivo using magnetism can effectively prevent vascular restenosis. | (Polyak et al., 2016) |
| Antisense RNA against platelet derived growth factor β (PDGF-β) | Chitosan NP carrying antisense RNA against platelet derived growth factor β (PDGF-β) | Male New Zealand rabbits (2.5 kg) | Rabbit iliac artery balloon injury | 1. Nontoxicity; 2. Good biodegradability; 3. Favorable biocompatibility; 4. Sustained release | Chitosan nanoparticles loaded with antisense RNA against PDGF-β inhibited the proliferation of VSMCs and the expression of PDGF-β mRNA, thereby reducing intimal hyperplasia and achieving the prevention of vascular restenosis. | (Xia et al., 2013) |
| Dexamethasone (DEX) | PEG–PLA–PEG triblock copolymers coated dexamethasone | —— | —— | 1. High loading efficiency; 2. Improved drug water solubility; 3. Slow release | Dexamethasone-carrying PEG-PLA-PEG nanoaggregates inhibited the proliferation of VSMCs better than dexamethasone alone. | (Park and Yoo, 2006) |
| RVX-208 (RVX) | RVX-loaded platelet membrane-coated nanoclusters (RVX-PM-NC) | Male Sprague‒Dawley rats (300~350 g) | Rat carotid artery balloon injury | 1. Excellent platelet targeting; 2. Good ROS targeting; 3. Size tunability; 4. Favorable biodistribution | RVX-PM-NC has the characteristics of targeting the damaged site of blood vessels, targeting the release of ROS, and reducing the size at the targeted location. In vivo experiments demonstrated that RVX-PM-NC significantly inhibited intimal hyperplasia. | (Zhao et al., 2021b) |
| Hep;  Rapamycin (RAP);  Cluster of differentiation 34 (CD34) antibody;  SB431542;  Necrostain-1 | Loading of PLGA NPs carrying different drugs into hydrogels | Male Sprague‒Dawley rats (6~8 weeks) | Rat inferior vena cava patch venoplasty | 1. Excellent biodegradability; 2. Multidelivery ability; 3. Favorable biocompatibility | PLGA NPs containing different drugs are loaded onto the three-layer hydrogel for multiple NP delivery, which can release drugs in layers. The three-layer hydrogel containing different drugs significantly inhibited intimal hyperplasia, VSMCs proliferation and thrombus formation. | (Wei et al., 2022) |
| Rapamycin (RAP) | RAP-containing pH and ROS dual-responsive nanoparticles (TAOCD) modified by a peptide (KLWVLPKGGGC) targeting type IV collagen (Col -IV) | Male Sprague‒Dawley rats (400~450 g); Male C57BL/6J mice (18~22 g) | Rat carotid artery balloon injury | 1. Good targeting; 2. Dual responsiveness; 3. Good biosafety | TAOCD NPs can prevent vascular restenosis with passive targeting of PH and ROS and active targeting of Col-IV, which is a triple targeting to prevent vascular restenosis. Moreover, TAOCD can significantly inhibit the proliferation and migration of VSMCs. | (Zhang et al., 2020) |
| Endothelium-protective epigenetic inhibitor (JQ1) | Nanoclusters formed by multiple PAMAM-polyvalerolactone (PAMAM-PVL) ultrasmall single-molecule NPs passing through the platelet membrane wrapped with loaded JQ1 (PAMAM-PVL-PM-JQ1 NPs) | Male Sprague‒Dawley rats (~400 g) | Rat carotid artery balloon injury | 1. Excellent targeting; 2. Promotion of intima reendothelialization; 3. Easy surface modification; 4. Easy size control | PAMAM-PVL-PM-JQ1 NPs not only target the site of vascular injury but also inhibit intimal hyperplasia while protecting intima reendothelialization. | (Wang et al., 2018) |
| Short interfering RNA (siRNA) | Cell penetrating peptides (CPPs) modified neutral liposomes | —— | —— | 1. Improved siRNA encapsulation efficiency; 2. Low cytotoxicity; 3. Strong cell binding | The gene transfection ability of LNPs after modification with CPPs was enhanced. | (Fisher et al., 2017) |
| Sirolimus (SIR);  Propolis | Lipid polymer-hybridized PLGA NPs loaded with SIR or propolis (LPHNSs) | —— | —— | 1. Both lipid layer and polymer core; 2. Simple preparation; 3. Strong stability | LPHNSs loaded with either SIR or propolis inhibited the proliferation of VSMCs, and propolis LPHNSs were nontoxic. PLGA NPs hybridized with lipid polymers had longer drug release retardation time and significantly lower drug burst release. | (Jc Bose et al., 2016) |
| Vascular endothelial growth factor (VEGF) | DNA-gelatin magnetic nanospheres with VEGF plasmids | Male New Zealand white rabbits (3.0~4.0 kg) | Rabbit femoral artery balloon injury | 1. Improved DNA stability and transfection efficiency; 2. Magnetic targeting; 3. Good biological safety; 4. Long duration of action | Magnetic DNA microspheres significantly improved DNA stability, gene transfection efficiency and targeting specificity and promoted exogenous VEGF overexpression in vivo. | (Zhang and Qu, 2016) |
| Aspirin;  Probucol | NPs of quaternary ammonium salt-modified chitosan (HACC) loaded with aspirin and probucol | —— | —— | 1. Delivery of both hydrophobic and hydrophilic drugs; 2. Sustained release; 3. Excellent water solubility | HACC NPs can release both hydrophobic and hydrophilic drugs and can carry large numbers of them. | (Liu and He, 2015) |
| Tissue-type plasminogen activator gene (t-PA) | Albumin NPs loaded with t-PA gene plasmid cross-linked with albumin ultrasound microbubbles. | Male healthy dogs (40~45 kg) | Dog coronary bypass | 1. Good biocompatibility and biodegradability; 2. Nonimmunogenicity; 3. Non-cytotoxicity; 4. Ultrasound targeting | The use of ultrasound allowed targeted delivery of albumin nanoparticles loaded with the t-PA gene to the site of the intervention, thereby preventing vascular restenosis. | (Ji et al., 2014) |
| Vascular endothelial growth factor (VEGF)  Paclitaxel (PTX) | Bilayered PLGA NPs containing VEGF in the outer layer and PTX in the inner core (VEGF/PTX NPs). | Mini-swine (approximately 35 kg) | Porcine coronary artery stent implantation | 1. Sequential drug release; 2. Excellent biodegradability; 3. Gene-drug combination therapy | VEGF/PTX NPs enabled sequential drug release, promoting intima re-endothelialization while inhibiting VSMC proliferation. | (Yang et al., 2013) |
| Dexamethasone (DEX) | Glycoprotein Ib alpha (GPIbα)-modified PLGA NPs loaded with DEX (GPIbα-DEX-PLGA NPs) | —— | —— | 1. Mimicry of platelet targeting; 2. Good biodegradability; 3. Sustained release; 4. Strong adhesion | GPIbα-DEX-PLGA NPs have a similar targeting effect to platelets and can target and adhere to the site of vascular injury, thereby preventing vascular restenosis. | (Kona et al., 2012) |
| Honokiol (HNK) | HNK-loaded LNPs | —— | —— | 1. Excellent biodegradability; 2. Sustained release; 3. High encapsulation rate | Dimyristoyl phosphatidylcholine (DMPC) liposomes could be effectively loaded with HNK, and liposome-encapsulated HNK inhibited the proliferation of VSMCs better than HNK alone. | (Chen, 2009) |
| Antisense RNA against monocyte chemotactic protein-1 (anti-MCP1) | anti-MCP1 encapsulated into PLGA NPs | New Zealand White rabbits (2.0~2.5 kg) | Rabbit carotid artery catheter injury | 1. Excellent biocompatibility and biodegradability; 2. Gene delivery | PLGA NPs are suitable for intravascular site-specific gene therapy and can realize gene transfection. Moreover, PLGA NPs carrying anti-MCP1 significantly inhibited intimal hyperplasia and inflammatory response. | (Yang et al., 2008) |
| —— | Nanosuspensions of alendronate-gallium and alendronate-gadolinium (AGA-AGD) | Male Sabra rats (350~420 g) | Rat carotid artery balloon injury | 1. No additives; 2. Systemic delivery; 3. Excellent biocompatibility and biodegradability | AGA-AGD nanosuspensions inhibited intimal hyperplasia, prevented restenosis, and are biodegradable. | (Epstein et al., 2007) |
| Alendronate (ALN) | PLGA NPs loaded with ALN (ALN-PLGA NPs) | New Zealand White rabbits (2.5~3.5 kg) | Hypercholesterolemic rabbit carotid artery balloon injury | 1. Nontoxicity; 2. Targeting of monocytes and macrophages; 3. Excellent biodegradability and biocompatibility | ALN-PLGA NPs prevented neointimal formation by targeted depletion of monocytes and reduction of arterial macrophages, thereby preventing vascular restenosis. | (Cohen-Sela et al., 2006) |
| AGL-2043 | AGL-2043-loaded PLGA NPs (AGL-PLGA NPs) | Male Sabra rats (350~420 g); Juvenile domestic swine (20~25 kg) | Rat carotid artery balloon injury; Pig coronary arteries stent implantation | 1. Excellent biodegradability and biocompatibility; 2. Sustained release | AGL-PLGA NPs inhibited the proliferation and migration of VSMCs as well as intimal hyperplasia, preventing vascular restenosis. | (Banai et al., 2005) |
| Pitavastatin | Pitavastatin-loaded PLGA NPs | Rats | Rat carotid artery balloon injury | 1. Sustained release; 2. Good biodegradability; 3. High biosafety | Pitavastatin promoted endothelial progenitor cell proliferation through PI3K signaling, and PLGA NPs loaded with pitavastatin promoted endothelial progenitor cell proliferation, thereby accelerating vascular endothelial repair. | (Liu et al., 2017a) |
| Platelet-derived growth factor β-receptor antisense (PDGFβR-AS) | PLGA NPs loaded with PDGFβR-AS (PDGFβR-AS NPs) | Male Sabra rats (350~420 g) | Rat carotid artery balloon injury | 1. Excellent biocompatibility and biodegradability; 2. High encapsulation rate; 3. Good slow-release performance | PDGFβR-AS NPs inhibited VSMC proliferation and intimal hyperplasia to prevent vascular restenosis. | (Cohen-Sacks et al., 2002) |
| Smad3 shRNA (shSmad3) | Polyethylene glycol-graft-polyethylenimine derivative (PEG-Et 1:1) loaded with shSmad3 | New Zealand White rabbits (3.0~3.5 kg) | Rabbit carotid artery balloon injury | 1. Reduced cytotoxicity; 2. Increased gene transfection efficiency; 3. Nonviral gene vectors | PEG modification improved the gene transfection efficiency and reduced the cytotoxicity of PEI-Et, and PEG-Et loaded with shSmad3 inhibited intimal thickening to prevent vascular restenosis. | (Wang et al., 2019) |
